# Zinc oxide end-capped Fe_3_O_4_@mSiO_2_ core-shell nanocarriers as targeted and responsive drug delivery system for chemo-/ions synergistic therapeutics

**DOI:** 10.1080/10717544.2019.1642419

**Published:** 2019-07-25

**Authors:** Minchao Liu, Xiangyu Sun, Zhihui Liao, Yahui Li, Xiaoliang Qi, Yuna Qian, Hicham Fenniri, Ping Zhao, Jianliang Shen

**Affiliations:** a School of Ophthalmology and Optometry, School of Biomedical Engineering, Wenzhou Medical University, Wenzhou, China;; b School of Chemistry and Chemical Engineering, Guangdong Pharmaceutical University, Guangzhou, China;; c Wenzhou Institute, University of Chinese Academy of Sciences, Wenzhou, China;; d Department of Chemical Engineering, Northeastern University, Boston, MA, USA;; e Department of Bioengineering, Northeastern University, Boston, MA, USA;; f Department of Chemistry and Chemical Biology, Northeastern University, Boston, MA, USA

**Keywords:** Magnetic mesoporous silica nanoparticles, ZnO, daunomycin, pH-responsive, synergistic effects

## Abstract

Multifunctional core-shell nanocarriers based on zinc oxide (ZnO)-gated magnetic mesoporous silica nanoparticles (MMSN) were prepared for cancer treatment through magnetic targeting and pH-triggered controlled drug release. Under an external magnetic field, the MMSN could actively deliver chemotherapeutic agent, daunomycin (DNM), to the targeted sites. At neutral aqueous, the functionalized MMSN could stably accommodate the DNM molecules since the mesopores were capped by the ZnO gatekeepers. In contrast, at the acid intercellular environment, the gatekeepers would be removed to control the release of drugs due to the dissolution of ZnO. Meanwhile, ZnO quantum dots not only rapidly dissolve in an acidic condition of cancer cells but also enhance the anti-cancer effect of Zn^2+^. An *in vitro* controlled release proliferation indicated that the acid sensitive ZnO gatekeepers showed well response by the ‘on-off’ switch of the pores. Cellular experiments against cervical cancer cell (HeLa cells) further showed that functionalized MMSN significantly suppressed cancer cells growth through synergistic effects between the chemotherapy and Zn^2+^ ions with monitoring the treatment process. These results suggested that the ZnO-gated MMSN platform is a promising approach to serve as a pH-sensitive system for chemotherapies delivery and Zn^2+^ controlled release for further application in the treatment of various cancers by synergistic effects.

## Introduction

In recent decades, nanomaterials and nanoparticles (NPs) served as drug delivery systems have been apace developed to improve cancer diagnosis and treatment while with minimum side effects of anti-cancer drugs in the health tissues (Gomes et al., 2014; Cheng et al., 2017c; Liang et al., 2018). The encapsulation of drugs within nanocarriers holds the promise to increase the accumulation of therapeutic agents in the targeted tissues due to enhanced permeability and retention (EPR) effects decrease the side effects and simultaneously increase the efficacy of cancer therapeutic (Han et al., 2015; Kanamala et al., 2016; Liu et al., 2017). To date, a variety of nanocarriers, including lipids, polymers, viruses and even inorganic NPs, has been developed as vehicles for drug delivery applications (Kozlova et al., 2012; Wang et al., 2014, 2013; Liu et al., 2016b, 2019; Cheng et al., 2017a). Among these nanocarriers, mesoporous silica nanoparticles (MSNs) have been extensively investigated as promising drug-delivered carriers due to their high surface area, good biocompatibility, excellent water dispersibility, well-defined pore size and easily modifiable surface (Cho & Borgens, 2010; Zhao et al., 2015; Chang et al., 2016; Cheng et al., 2017b). Especially, in recent decades, magnetic mesoporous silica nanoparticles (MMSN) have drawn growing interest for the multifunctions of magnetic resonance imaging (MRI), targeted drug delivery, magnetic enrichment and purification (Kievit et al., 2011; Chang et al., 2012; Shen et al., 2015). Due to the core-shell structures, MMSN can allow the single nanocomposite to exert multifunctions including targeted delivery ability, therapy and imaging (Zhao et al., 2014; Verma et al., 2015).

In using MMSN as drug delivery vehicles, extensive efforts have been devoted to the fabrication of stimuli-responsive drug releasing system. A number of capping scaffolds, such as inorganic NPs (Giri et al., 2005; Liu et al., 2018b), polymers (Zhu et al., 2011; Guo et al., 2014), organic molecules (Zeng et al., 2017), and biomacromolecules (Patel et al., 2008; Bernardos et al., 2009; Wu et al., 2013), functionalized MSNs gatekeepers to realize the controlled release of drugs in response to endogenous stimuli (e.g. pH, enzymes, redox and specific analysts) and exogenous stimuli (e.g. temperature, light, ultrasound, electric field and magnetic fields) (Cotí et al., 2009; Luo et al., 2011; Yang et al., 2012; Coll et al., 2013; Mura et al., 2013).

As to the pH stimuli, it is well known that low pH is a common feature in tumor and inflammatory tissues. In contrast to the neutral environment of the blood/normal tissues (pH 7.4), the early endosomes and late endosomes/lysosomes in the tumor cells are at approximately pH 6.0 and pH 5.0, respectively (Miyata et al., 2008). This allows nanocarriers to respond to pH differences between tumors and normal tissues due to their different physiological environments. Various inorganic/organic nanomaterials were employed as pH-stimulate controlled drug release systems.

Among them, ZnO quantum dots (QDs), which benefited from their low cost, ease of synthesis, excellent biocompatibility and ease of functionalization, were payed special attention as gatekeepers to construct gated MSN delivery system. For instance, Ye et al. (2016) prepared ZnO-Gd-DOX (DOX: doxorubicin) nanoplatform, which possesses low toxicity, excellent biocompatibility, easy preparation process and pH sensitivity and can sustainably release encapsulated DOX into the acid environment of tumors. Wang et al. (2015) prepared a multifunctional nano-theranostic agent with UCNPs (NaYF_4_: 20%Yb^3+^, 2%Er^3+^/NaGdF_4_: 2%Yb^3+^) as the core for the UCL/CT/MRI trimodality imaging, and a mesoporous silica layer as the outer shell with ZnO as gatekeeper for pH-triggered drug delivery. ZnO QDs are stable at the physiological environment, but undergo a rapid dissolution into zinc ions in an acidic condition of cancer cells. Furthermore, stable ZnO NPs exhibited low cytotoxicity in the physiological environment, while ZnO NPs dissolve into zinc ions (Zn^2+^) in cancer cells and cause great toxicity when they reach a certain concentration (Zhang et al., 2013; Qiu et al., 2017). Zinc ions can produce reactive oxygen species (ROS) to kill cancer cells, and the drug can be released to achieve the combined treatment effect (Zheng et al., 2017).

In this paper, Fe_3_O_4_@mSiO_2_ core-shell nanomaterials were fabricated as drug-loading hosts to deliver chemotherapeutical agent daunomycin (DNM) with ZnO used as gatekeepers. Due to the dissolution of ZnO in an acidic environment, it is expected that the drug delivery vehicle blocks pores in a neutral environment of blood and normal cells while opens pores in an acidic environment of cancerous tumors to control the chemo-drug release (Cai et al., 2016). As mentioned, ZnO QDs not only rapidly dissolve in an acidic condition of cancer cells but also enhance the anti-cancer effect of Zn^2+^. The system can be triggered to release drug in response to acidic pH in tumors and improved the synergistic therapeutic indexes.

## Materials and methods

### General materials and reagents

Iron (II) chloride tetrahydrate (FeCl_2_·4H_2_O), iron (III) chloride hexahydrate (FeCl_3_·6H_2_O) and ammonia solution (NH_3_·H_2_O) were purchased from Shanghai Aladdin Regent Co., Ltd. (Shanghai, China). Zinc acetate dehydrate (Zn(OAc)_2_·2H_2_O), isopropanol, tetraethyl orthosilicate (TEOS) and methanol (CH_3_OH), 3-aminopropyltriethoxysilane (APTES) (reagent grade, >98%) were purchased from Sigma-Aldrich. Cell Cycle and Apoptosis Analysis Kit, Prussian Blue-Neutral Red Staining Kit, and 3-(4,5-Dimethylthiazol-2-yl)-2,5-diphenyltetrazolium bromide (MTT) were obtained from Beyotime Biotech (Shanghai, China). Trypsin-EDTA solution and penicillin-streptomycin solution, fetal bovine serum (FBS) and DMEM medium were purchased from Gibco Life Technologies. All the other chemicals were of analytical grade, and Millipore water (18.2 MΩ) was used throughout the experiments.

### Experimental methods

#### Preparation of Fe_3_O_4_@mSiO_2_

Fe_3_O_4_ NPs were prepared according to a reference procedure (Liu et al., 2016a). Fe_3_O_4_@mSiO_2_ core-shell nanocomposites were fabricated via the sol-gel process of hydrolysis and condensation of TEOS (Jaeyun et al., 2006). Typically, 200 mg of dried Fe_3_O_4_ NPs were dissolved in 100 mL 0.1 M of dilute hydrochloric acid, and then 60 mL water and 140 mL ethanol were added at 37 °C. Subsequently, 0.2 mL TEOS was added dropwise to the above mixture solution under vigorous stirring. After 4 h of stirring, 0.6 mL of ammonia solution was added dropwise to initiate the TEOS hydrolysis and continued to be stirred at room temperature for 24 h under vigorous stirring. The NPs were collected through magnetic separation and washed several times with a 1:1 ethanol:water mixture to remove the residual reactants. Next step, in a mixed solution of 350 mg of cetyltrimethylammonium bromide (CTAB) in ethanol and deionized water, 1.5 mL of 28% aqueous ammonia was then added to the mixture, followed by sonication in a sonicator for 10 min. After stirring at room temperature, 0.2 mL of TEOS was slowly added to the round bottom flask and the reaction was continued for 6 h. Separated with a magnet and repeatedly washed with water and ethanol, and 100 mg of Fe_3_O_4_@mSiO_2_-CTAB magnetic NPs were dispersed in 50 mL of ammonium nitrate-ethanol solution (concentration of 10 mg/mL), uniformly dispersed by ultrasonic, and heated to reflux at 80 °C. The stirring speed was controlled at 200 rpm for 6 h, and then it was washed several times with ethanol and water and then repeated twice to achieve complete removal of CTAB. Fe_3_O_4_@mSiO_2_ was obtained.

#### Synthesis of ZnO QDs

ZnO QDs were synthesized using the method reported previously with slight modification (Zhang et al., 2011). Zinc acetate dihydrate (0.22 g, 1 mmoL) was dissolved in hot isopropanol (80 mL) with vigorous stirring and then diluted with isopropyl alcohol to a total volume of 920 mL. In a separate vessel, NaOH solution (80 mL, 2 × 10^−2 ^mol^ ^L^−1^) was dissolved in refluxing isopropanol (10 mL) and the solution was then cooled in an ice bath. The NaOH solution was quickly injected into a mixed solution of isopropanol. The mixture was stirred for 6 h to grow QDs, and the resulting clear dispersion of QDs showed green emission under UV lamp irradiation. Finally, ZnO QDs were precipitated using hexane as a non-solvent.

#### Determination of drug loading content and drug entrapment efficiency

The loading content and entrapment efficiency of DNM were measured by UV-3150 spectrophotometer (Shimadzu, Japan) following our previous protocol (Liu et al., 2018a). Generally, DNM was firstly dissolved in distilled water and stored at 4 °C before using. For the drug loading process, 10 mg freeze-dried Fe_3_O_4_@mSiO_2_ NPs were added in 10 mL DNM solution for 24 h. Afterward, the mixtures were recovered by magnetic separation followed by centrifugation. The concentration of DNM of the obtained supernatant was determined by UV-3150 spectrophotometer (Shimadzu, Japan) at 480 nm, and the entrapment efficiency and DNM loading capacity were calculated by Equations (1) and (2) as follows, respectively:

(1) $$\text{DNM}\;\text{entrapment}\;\text{efficiency}\;\left(\%,w/w\right)=\frac{\text{Weight}\;\text{of}\;\text{DNM}\;\text{nanoparticles}}{\text{Weight}\;\text{of}\;\text{DNM}\;\text{used}\;\text{in}\;\text{formulation}}\times 100\%,$$

(2) $$\text{DNM}\;\text{loading}\;\text{capacity}\;\left(\%,\;w/w\right)=\frac{\text{Weight}\;\text{of}\;\text{DNM}\;\text{in}\;\text{nanoparticles}}{\text{Weight}\;\text{of}\;\text{DNM}\;\text{used}\;\text{in}\;\text{recovered}}\times 100\%.$$

#### ZnO QDs coating on MSN

Five milligram of nano-zinc oxide was added to a beaker containing isopropanol and heated in a water bath at 85 °C. When the isopropyl alcohol was about to evaporate, 5 mL of distilled water was added to the beaker to continue heating, and the water was evaporated to dryness, and then rinsed several times with distilled water. Then, zinc oxide was added to the drug-loaded magnetic liquid, and after a slight shock, it was placed in a shaker and allowed to stand overnight. Finally, the Fe_3_O_4_@mSiO_2_-DNM-ZnO NPs were obtained by magnetization.

#### *In vitro* drug release study

The in vitro release of DNM from Fe_3_O_4_@mSiO_2_-DNM-ZnO NPs was determined by dissolving 10 mg Fe_3_O_4_@mSiO_2_-DNM-ZnO NPs in 5 mL PBS (0.2 mol L^−1^, pH 7.4 or 5.6) and shaken (100 rpm) at 37 °C. At desired intervals, the release medium (2 mL) was withdrawn and replaced with an equal volume of fresh medium. DNM concentrations of the release medium were measured by UV-3150 spectrophotometer (Shimadzu, Japan). The release experiments were performed in triplicate.

#### Biocompatibility research

HeLa cells were seeded in 96-well plates at a density of 7 × 10^3^ cells per well. After culture for 24 h, the cells were incubated with new medium containing Fe_3_O_4_@mSiO_2_-ZnO NPs at different concentrations (6.25, 12.5, 25, 50 and 100 μg/mL). Cells without treatment were used as control. After further incubated for 48 h, the cell viability was evaluated using MTT Cell Proliferation and Cytotoxicity Assay Kit. All experiments were conducted in thrice.

#### Prussian blue staining

HeLa cells were seeded in 12-well plates and then incubated with Fe_3_O_4_@mSiO_2_-ZnO NPs at a concentration of 50 μg/mL for 6 h. After incubation, the medium was removed, and the cells were washed three times with PBS. Following fixation for 20 min in 4% paraformaldehyde at room temperature, the cells were incubated in a Prussian blue staining solution (1:1 mixture of 5% potassium ferrocyanide (II) trihydrate solution and 5% HCl) for 30 min before being counterstained with neutral red. The cells were then washed three times with PBS and observed by light microscopy.

#### Cell migration inhibition study by scratch wound assay

Migration of HeLa cells was determined using a scratch wound migration assay. HeLa cells were seeded in 6-well plates at a density of 1 × 10^4^ cells/well. When achieving 90% of confluency after overnight, a scratch was made in cell culture using a sterile 200-ll pipet tip and washed twice to remove attached cells. The scratched monolayer cultures were photographed under an inverted microscope for a total period of 48 h. A defined area of the wound was then measured to determine cell migration.

#### Magnetic targeting study

HeLa cells were seeded in a 60 mm culture dish at a density of 5.0 × 10^4^ and were cultured for 24 h. Fe_3_O_4_@mSiO_2_-DNM-ZnO NPs with a concentration of 50 μg/mL was added to the culture dish. In order to carry out targeted research, a magnet (about 4 T) was placed under the petri dish and incubated for 24 h. At the bottom of the petri dish, the magnet region (targeted region) and the non-magnet region (non-targeted region) were observed, respectively, by optical microscope, and the cell morphology was photographed and observed.

#### Cell culture and MTT cell viability assay

Culture of HeLa cells was carried out using a previously reported method (Zhu et al., 2010). HeLa cell viability was assessed by 3-(4,5-dimethylthiazol-2-yl)-5-(3-cayboxymethoxyphenyl)-2-(4-sulfophenyl)-2H-tetrazolium (MTT) assay as described by Zhu et al. (2010) with some modifications. Briefly, HeLa cells were seeded at 7 × 10^3^ cells per well in 96-well plates and cultured for 48 h before exposure to free DNM, Fe_3_O_4_@mSiO_2_-DNM and Fe_3_O_4_@mSiO_2_-DNM-ZnO NPs at a serial concentration for 6 h. After exposure, 100 μL of MTT solution (0.5 mg mL^−1^) was added to each well. The cell was further incubated for 4 h at 37 °C. After removing medium carefully, the resulting formazan product was dissolved in DMSO (150 μL per well). The absorbance at 490 nm was recorded using a microplate reader (Perkin Elmer). The percentage (%) viability is calculated by the following equations:

$$\text{Cell}\;\text{vitality}\;\left(100\%\right)=\left(\frac{\text{ODdrug}-\text{ODblank}}{\text{ODcontrol}-\text{ODblank}}\right)\times 100.$$

OD drug is the absorbance of cells incubated with the different concentration of DNM or Fe_3_O_4_@mSiO_2_-DNM-ZnO NPs, OD blank is the absorbance of growth medium and OD control is the absorbance of cells incubated with growth medium.

#### Cell internalization studies

To visualize the cellular uptake of DNM with different treatments, the cells were seeded at a density of 1 × 10^5^ cells/mL in six-well culture plates. After that, the cells were co-cultured with free DNM or Fe_3_O_4_@mSiO_2_-DNM-ZnO NPs for 24 h. Afterward, the culture medium was removed, and cells were washed three times with PBS. The cells were observed by the IX 70 fluorescence microscope (Olympus, Japan).

#### Cell cycle phase distribution and apoptosis assay

HeLa cells seeded on the 6-well plates were treated with Fe_3_O_4_@SiO_2_-DNM-ZnO NPs, Fe_3_O_4_@SiO_2_-DNM NPs and free DNM (DNM concentration of 0.27 μg mL^−1^) and incubated in CO_2_ for 24 h at 37 °C. Cells treated with FBS-free culture medium served as control. At the end of incubation, adherent and non-adherent cells were trypsinized. Cells were harvested by centrifugation, washed twice with ice with cold PBS and fixed in 70% ethanol at 4 °C overnight. After 15 min incubation with 50 µL RNase A plus 450 µL propidium iodide (PI) in the dark, cells were subjected to cell cycle analysis using the flowcytometry (FACS Calibur, BD, USA), and the cell cycle distribution was analyzed by ModFit software.

For the quantitative analysis of apoptosis, HeLa cells (1 × 10^5^ cells/well) were seeded in 6-well plates. Then, cells were left untreated or were treated with free DNM and Fe_3_O_4_@SiO_2_-DNM-ZnO NPs. Afterward, cells were harvested and washed with PBS. Cellular apoptosis was detected using Annexin V-fluorescein isothiocyanate (FITC)/PI apoptosis detection kit. The stained cells were analyzed by flow cytometry within 1 h. Data analysis was performed using FlowJo software (Version 7.6.1) (Barnaby et al., 2011).

### Statistical analysis

All of the data were expressed as the mean ± SD. Differences between two groups were analyzed by a two-tailed Student’s *t* test. Differences with *P < .05 were considered statistically significant.

## Results and discussion

### Synthesis and characterization

**Figure 1.**
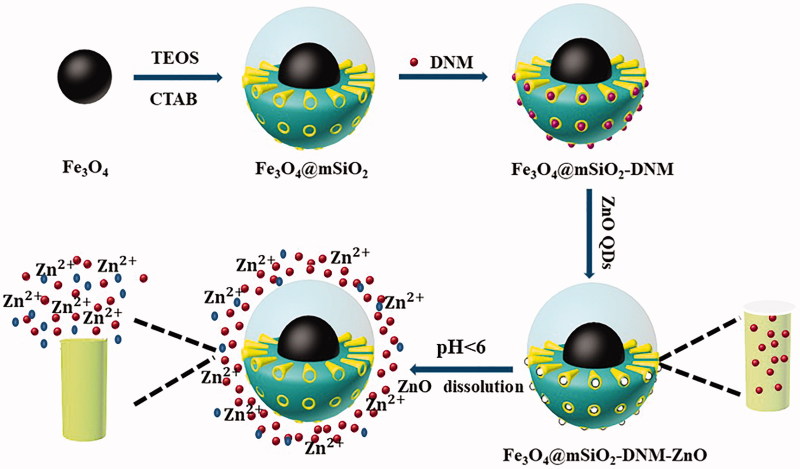
Schematic illustration for the construction of Fe_3_O_4_@mSiO_2_-DNM-ZnO NPs and the controlled release process.

The Fe_3_O_4_/SiO_2_ core-shell NPs were fabricated using the modified Stöber method (Slowing et al., 2008; Yang et al., 2015). In this synthesis method, the organosilane precursor TEOS interacts with a positively charged CTAB template that assembles into tubular micelles in water. The condensation occurs in the presence of sodium hydroxide, which is a basic catalyst for the hydrolysis of TEOS (Harris et al., 1990). Granular micelles are formed by condensation of silanol groups in the template CTAB (Baek et al., 2015). The surfactant is then removed by extraction with ammonium nitrate-ethanol solution, which dissolves the CTAB micelles (Shen et al., 2016). By using this preparing method, the fabricated Fe_3_O_4_/SiO_2_ system has a highly ordered porous structure with satisfied size and morphology. As shown in this controlled release system (Figure 1), ZnO acts as gatekeepers and can be manipulated by pH stimulation. When these gatekeepers are stimulated by the environment, these gatekeepers allow the payloads to be released from storage into a specific environment. In this study, the construction of this drug-loading system gave the possibility of pH response characteristics of these nanocarriers. This results in faster drug release at acidic pH, but it is interesting to note that the ZnO QDs coating remains relatively stable under physiological conditions.

**Figure 2.**
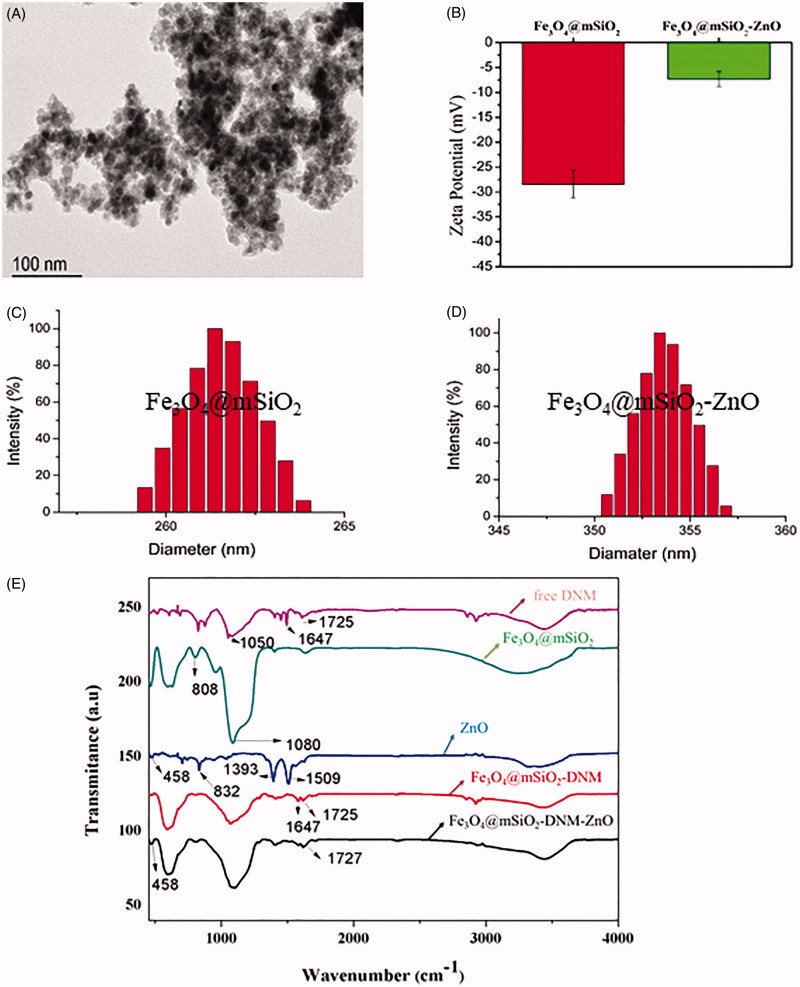
TEM image of (A) Fe_3_O_4_@mSiO_2_-DNM-ZnO NPs and (B) zeta potentials of Fe_3_O_4_@mSiO_2_ and Fe_3_O_4_@mSiO_2_-ZnO nanoparticles and the size distribution of (C) Fe_3_O_4_@mSiO and (D) Fe_3_O_4_@mSiO_2_-ZnO nanoparticles, and (E) FTIR spectra of free DNM, Fe_3_O_4_@mSiO_2_, ZnO, Fe_3_O_4_@mSiO_2_-DNM and Fe_3_O_4_@mSiO_2_-DNM-ZnO.

The size and morphology of Fe_3_O_4_@SiO_2_-ZnO NPs were studied by JEM-2010 HR transmission electron microscope (JEOL, Japan). As shown in Figure 2(A), Fe_3_O_4_@SiO_2_-ZnO NPs were spherical with an average diameter of 20 nm, and the outer layer in particles was clearly distinguishable. Size distribution and zeta potentials of the particles were obtained on a Malvern Zetasizer Nano ZS90 instrument (Malvern, UK). Figure 2(B) shows zeta potentials of Fe_3_O_4_@mSiO_2_ and Fe_3_O_4_@mSiO_2_-ZnO NPs, which varies from –27.43 ± 2.76 to –7.3 ± 1.52, respectively. The changes on potential further confirmed the successful fabrication of ZnO QDs-conjugated Fe_3_O_4_@mSiO_2_ NPs. Figures 2(C,D) showed the particle size distribution histogram of Fe_3_O_4_@mSiO_2_ NPs and Fe_3_O_4_@mSiO_2_-ZnO NPs, respectively. Fe_3_O_4_@mSiO_2_ NPs showed a narrow size distribution ranging from 240 nm to 276 nm with an average particle size diameter of 260 nm in Figure 2(C). Furthermore, the size distribution of Fe_3_O_4_@mSiO_2_-ZnO NPs was from 350 nm to 356 nm with an average particle size diameter of 353 nm (Figure 2(D)), which evidenced that ZnO QDs-conjugated Fe_3_O_4_@mSiO_2_ NPs were synthesized successfully.

FTIR spectra were recorded on an Equinox 55 Fourier transformation infrared spectrometer (Bruker, Germany); FTIR spectra of free DNM, Fe_3_O_4_@mSiO_2_, ZnO, Fe_3_O_4_@mSiO_2_-DNM and Fe_3_O_4_@mSiO_2_-DNM-ZnO were shown in Figure 2(E). In the IR spectrum of free DNM, a pair of bands at 1050 cm^−1^, and another pair of bands at 1647 cm^−1^ and 1725 cm^−1^ were attributed to the characteristic C–N and N–H stretching, respectively. The characteristic peaks of the Fe_3_O_4_@mSiO_2_ NPs appeared at 808 cm^−1^ and 1080 cm^−1^, which could be assigned to the Si–O bond stretching vibration and the Si–O–Si vibration, respectively (Kostiv et al., 2017). In the spectrum of ZnO, peaks at 458 cm^−1^, 832 cm^−1^, 1393 cm^−1^ and 1509 cm^−1^ could be attributed to Zn–O stretching. For the Fe_3_O_4_@mSiO_2_-DNM NPs, a new strong absorption peak appeared at 1647 cm^−1^ and 1725 cm^−1^, corresponding to the stretching vibrations of C–N groups and N–H, respectively. This demonstrated that DNM was modified successfully on the surface of Fe_3_O_4_@mSiO_2_ NPs. Two strong absorption peaks appeared at 458 cm^−1^ and 1725 cm^−1^ for Fe_3_O_4_@mSiO_2_-DNM-ZnO NPs, which could be assigned to the Zn–O asymmetric stretching and –NH_2_ symmetric vibrations, respectively (Yuan et al., 2010). The FTIR spectrum of Fe_3_O_4_@mSiO_2_-DNM-ZnO NPs showed the characteristic absorption bands of both ZnO and Fe_3_O_4_@mSiO_2_-DNM NPs, confirming successful gating and drug loading of the designed MMSN NPs.

### *In vitro* DNM loading and triggered release

**Figure 3.**
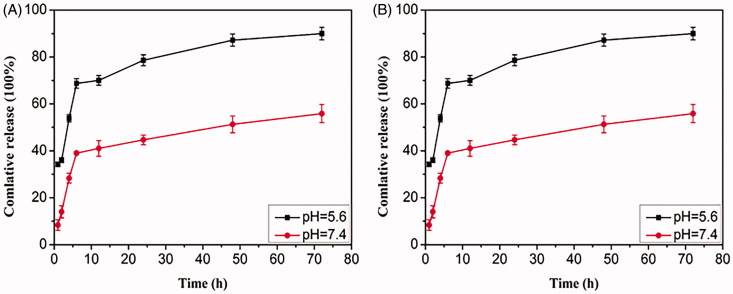
pH-responsive release profiles of DNM from (A) Fe_3_O_4_@mSiO_2_-DNM and (B) Fe_3_O_4_@mSiO_2_-DNM-ZnO NPs delivery systems at pH 7.4 and 5.6 at 37 °C.

DNM was loaded into the prepared Fe_3_O_4_@mSiO_2_ NPs and the drug loading and releasing property were evaluated by measuring the concentration of unbound drug at the absorbance of 490 nm. The loading efficiency and entrapment efficiency of Fe_3_O_4_@mSiO_2_-DNM NPs were calculated to be 3.76 ± 0.11% and 25.06 ± 0.77%, respectively. The drug releasing behaviors of the Fe_3_O_4_@mSiO_2_-DNM NPs were assessed at 37 °C under different pH values of 5.6 and 7.4, which imitated late endosome and normal tissue, respectively. As shown in Figure 3, the release of DNM from the non-coated particles presents two profiles at both pHs (Figure 3(A)), with a first phase characterized by a clear burst release from 1 h until 4 h. After this second phase, DNM is almost totally released by a relatively slow release until 48 h. In contrast to non-coated Fe_3_O_4_@mSiO_2_ NPs, a more controlled release at pH 7.4 and pH 5.6 was obtained for ZnO coated Fe_3_O_4_@mSiO_2_ NPs as shown in Figure 3(B). Compared with the previous Fe_3_O_4_@mSiO_2_-DNM system, the present system showed the effect of zinc oxide coating in the release of DNM and highlight the pH-sensitive behavior of zinc oxide coating since their incubation in a slightly acidic physiological environment prompted a noticeable increase in the amount of detected drug in comparison with the Fe_3_O_4_@mSiO_2_-DNM-ZnO NPs incubated at a neutral physiological environment (Figure 3(B)). The rapid release of DNM is the result of the dissolution of zinc oxide, which resulted in the open of the pores at the surface of silica NPs. This study clearly shows that Fe_3_O_4_@mSiO_2_-DNM-ZnO NPs have pH-sensitive release characteristics, which is expected to minimize the premature release of cytotoxic drugs in the blood circulation and promote the release of active targets of acidic drugs.

### Cellular uptake of the Fe_3_O_4_@mSiO_2_-DNM-ZnO NPs study

**Figure 4.**
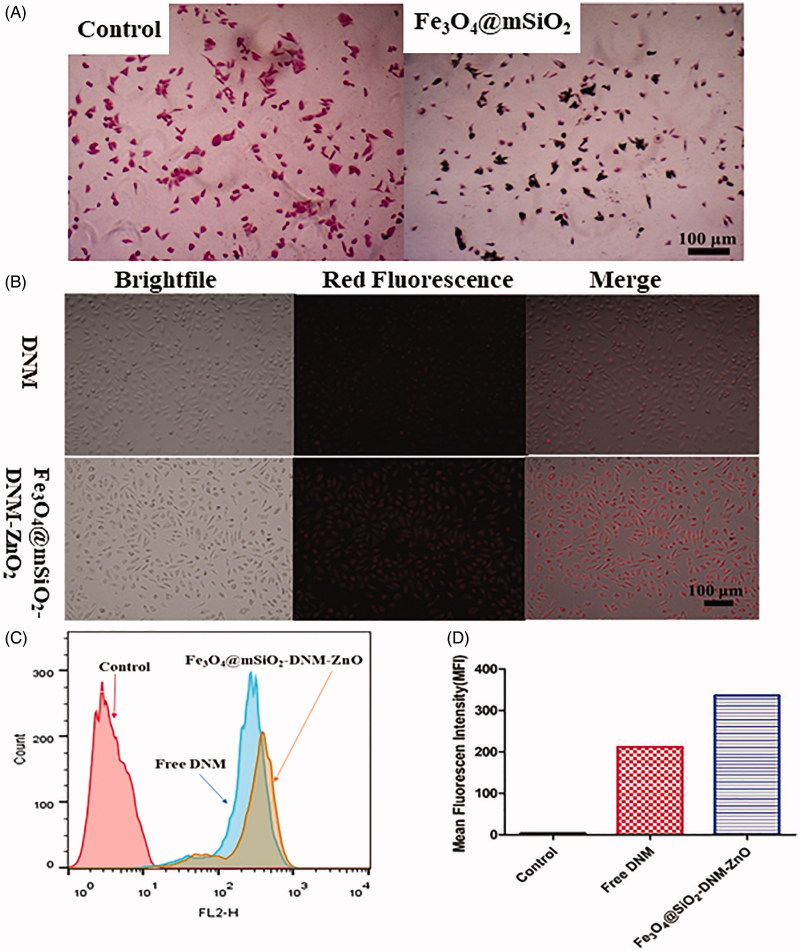
(A) Prussian blue staining of HeLa cells treated with the magnetic nanocarrier (50 μg/mL) or control cell media without the magnetic carrier for 6 h. (B) The cell internalization of free DNM and Fe_3_O_4_@mSiO_2_-DNMZnO NPs assessed by a fluorescence microscope. Quantitative analysis of the fluorescence intensity, (C) MFI flow cytometry analysis to HeLa cells and (D) representative histograms of DNM in different nanoparticles.

It is noted that effective cell internalization of nanocarriers plays a critical role in the delivery of drug into the target cells (Ding et al., 2012). To detect the presence of the studied Fe_3_O_4_@mSiO NPs in HeLa cells, as shown by Prussian blue staining in Figure 4(A), intracellular ‘iron’ was detected staining of cells which had been incubated with the magnetic nanocarrier, no significant blue staining was observed for the control cells. In contrast, substantial blue spots were observed incubated with Fe_3_O_4_@mSiO_2_ NPs in most of the cells. Most blue spots appear to be located around the cell membrane and throughout the cytoplasm, indicating that the uptake of magnetic nanocarriers by the cells is high.

The above results of the Prussian blue staining experiment proved that the MMSN vehicle could enter the cancer cells. On the basis of this success, we further researched whether the payload of this vehicle, the DNM drug efficiently entered the HeLa cells. Benefiting from its red fluorescence, the intracellular DNM drug can be effortlessly observed under a fluorescence microscope without additional markers. The cell uptake results of DNM in free form and MMSN loaded system were shown in Figure 4(B), red fluorescence was observed for both cells exposed to Fe_3_O_4_@mSiO_2_-DNM-ZnO NPs or free DNM, indicating that DNM could enter the cells efficiently at both states. Moreover, the merged pictures showed that the group of Fe_3_O_4_@mSiO_2_-DNM-ZnO NPs has a higher fluorescence intensity than that of the free DNM, which may be attributed to the EPR effect of the nanomaterials.

Flow cytometry analysis was performed to further research the endocytosis of Fe_3_O_4_@mSiO_2_-DNM-ZnO NPs by HeLa cells. Since DNM itself is fluorescent, no additional markers were used in the flow cytometry analysis and the fluorescence intensity directly corresponds to the amount of DNM internalized by the cells. As shown in Figure 4(C), cells without any DNM treatment were used as a negative control and showed only the auto-fluorescence of the cells. When the cells were treated with DNM or Fe_3_O_4_@mSiO_2_-DNM-ZnO NPs, the fluorescent signals in the cells were significantly increased. Both free DNM and Fe_3_O_4_@mSiO_2_-DNM-ZnO NPs were taken efficiently by cells while the latter behaving better than the former. As showed in Figure 4(D), the amount of cellular uptake of Fe_3_O_4_@mSiO_2_-DNM-ZnO NPs (MFI = 336) was high than that of DNM (MFI = 212). This result further evidenced the phagocytic function of HeLa cells to the Fe_3_O_4_@mSiO_2_-DNM-ZnO NPs, indicating that the designed MMSN vehicle is efficient in drug delivery.

### Magnetic targeting and cell-migration study

**Figure 5.**
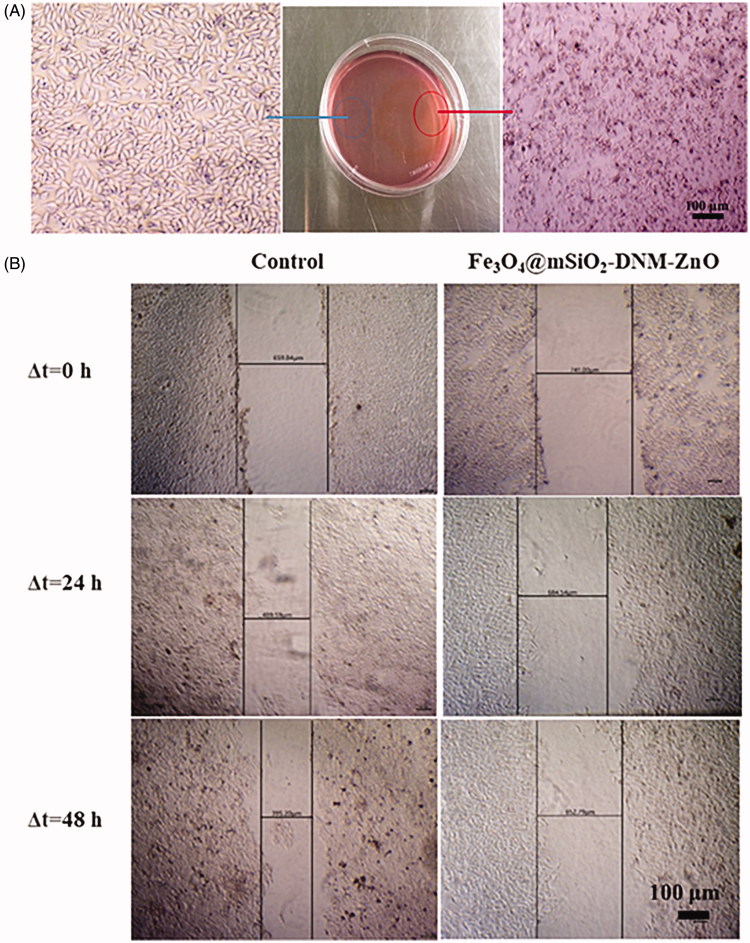
(A) Microscopy images of HeLa cells at the targeting area (red circle) and the control area (blue circle) after exposure to Fe_3_O_4_@mSiO_2_-DNM-ZnO NPs (with Fe_3_O_4_@mSiO_2_-DNM-ZnO NPs concentration of 50 μg/mL^–1^) for 24 h under an external magnetic field (about 4 T). (B) Analysis of cell migration for control and cells incubated with Fe_3_O_4_@mSiO_2_-DNM-ZnO NPs for 0 h, 24 h and 48 h by scratch wound assay.

An in vitro magnetic targeting experiment was designed and performed to examine the magnetic targeting property of the magnetic nanocarrier. As shown in Figure 5(A), after incubation, in the targeting area (red circle), the amorphous of the cells changes greatly and a majority of the cells were floating. In contrast, for the region where the magnetic field is much weaker (blue circle), most of the cells are in good shape, and the cell death is much less than the magnetic position, indicating that the Fe_3_O_4_@mSiO_2_-DNM-ZnO NPs could accumulate in the targeting area under a magnetic field. Therefore, these results demonstrated that the external magnetic field can significantly increase the local concentration of the Fe_3_O_4_@mSiO_2_-DNM-ZnO NPs, which means that Fe_3_O_4_@mSiO_2_ NPs can effectively transport the goods to the target area under an external magnetic field.

A wound-healing assay was performed to investigate the effect of Fe_3_O_4_@mSiO_2_-DNM-ZnO NPs on the migration of HeLa cells, and the scratched monolayer was photographed at 0, 24 and 48 h. The results were given in Figure 5(B), from which it could be clearly found that the wound healing was significantly suppressed by Fe_3_O_4_@mSiO_2_-DNM-ZnO NPs compared with that of the control cells. As is shown in Figure 5(B), compared to the control group, Fe_3_O_4_@mSiO_2_-DNM-ZnO NPs significantly decreased the repair rate of the wound. Our results showed that the minimal fraction of HeLa cells not committed to cell death did not display any capacity of closing wounds after incubation with the NPs.

### *In vitro* cytotoxicity study

**Figure 6.**
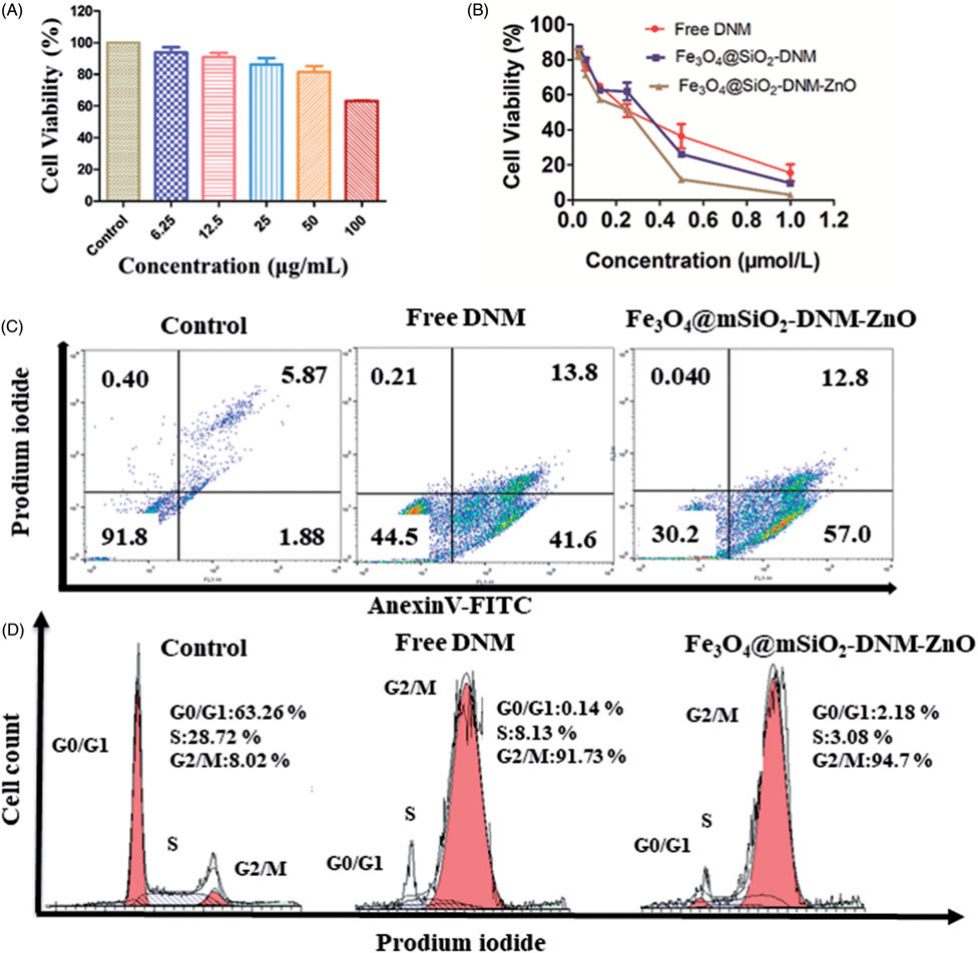
(A) HeLa cell viability under different concentrations of Fe_3_O_4_@mSiO_2_-ZnO NPs. (B) Cytotoxicity of free DNM, Fe_3_O_4_@mSiO_2_-DNM NPs and Fe_3_O_4_@mSiO_2_-DNM-ZnO NPs at various concentrations against HeLa cells. (C) FACS analysis of apoptosis in HeLa cells incubated with 0.27 μg/mL of Fe_3_O_4_@mSiO_2_-DNM-ZnO NPs measured using FITC-labeled Annexin V and PI. Representative dot plots showing the distribution of Annexin V and PI staining for control, free DNM and Fe_3_O_4_@mSiO_2_-DNM-ZnO NPs-treated cells. (D) Cell cycle status of the HeLa cell line after treatment with free DNM and Fe_3_O_4_@mSiO_2_-DNM-ZnO NPs for 24 h.

Biocompatibility is a necessary prerequisite to the design of a drug delivery vehicle. The MTT method is a general quantitative data describing the degradable cytotoxicity of biological materials. Here, we have performed the MTT method to test the viabilities of HeLa cells treated with Fe_3_O_4_@mSiO_2_-ZnO NPs for 48 h. As showed in Figure 6(A), with the concentration of Fe_3_O_4_@mSiO_2_-ZnO increasing from 6.25 μg/mL to 50 μg/mL, there was no substantial decrease of cell viabilities observed. The viabilities of HeLa cells kept over 81% when incubated with the Fe_3_O_4_@mSiO_2_-ZnO at the concentration up to 50 μg/mL. This result guaranteed that the designed Fe_3_O_4_@mSiO_2_-ZnO vehicle are biocompatible and can be harnessed in drug delivery among intracellular experiments. However, Fe_3_O_4_@mSiO_2_-ZnO NPs exhibited significant cytotoxic effects when the concentration of Fe_3_O_4_@mSiO_2_-ZnO NPs was as high as 100 μg/mL. This could be attributed to the cytotoxicity of Zn^2+^, which has been reported to have induction of ROS production, lipid peroxidation and DNA damage (Hanley et al., 2008; Xia et al., 2008; Deng et al., 2009).

To investigate the cytotoxicity of DNM loaded Fe_3_O_4_@mSiO_2_-ZnO NPs, cellular cytotoxicity assays of Fe_3_O_4_@mSiO_2_-DNM-ZnO NPs, Fe_3_O_4_@mSiO_2_-DNM NPs and free DNM against HeLa cells were performed and the results were revealed in Figure 6(B). From Figure 6(B), it is easy to find that there is a dose-dependent manner between the cell survival rates and all the studied drug delivery systems. Interestingly, Fe_3_O_4_@mSiO_2_-DNM-ZnO NPs have higher cellular inhibition than Fe_3_O_4_@mSiO_2_-DNM NPs. This could be attributed to the dissolution of ZnO under the tumor microenvironment, which resulted in the burst of DNM release in the acid intercellular environment and thus enhanced the efficiency of drugs. ZnO NPs were confirmed to be qualified as gatekeepers in this pH-triggered drug delivery vehicle. The release of the loaded drug would be controlled by controllable on-off of the mesopores for sustained delivery and high anti-cancer therapeutic efficiency.

Annexin V/PI double staining was used to detect apoptosis in HeLa cells. HeLa cells were treated with Fe_3_O_4_@mSiO_2_-DNM-ZnO NPs of an IC_50_ concentration (0.27 μg/mL) for 24 h. Figure 6(C) shows the degree of apoptosis caused by the Fe_3_O_4_@mSiO_2_-DNM-ZnO NPs delivery system as assessed by the fluorescence-activated cell sorting (FACS) analysis. The percentage of early and late apoptotic HeLa cells in the control group was 1.88% and 5.87%, respectively, and the percentage of apoptosis was the lowest in the three test treatments. In contrast, the group of free DNM caused 41.6% and 13.8% while Fe_3_O_4_@mSiO_2_-DNM-ZnO NPs caused 57.0% and 12.8% for early and late cell apoptosis, respectively. Fe_3_O_4_@mSiO_2_-DNM-ZnO NPs exhibit stronger capability of inducing cell apoptosis than free DNM, which in high accordance with the experimental results above.

According to the change of DNA content in cells, the cell cycle could be divided into three test points: G0/G1, S, and G2/M. If the DNA in cells is inhibited and damaged, the cell cycle will be blocked in a specific detection segment, we call G0/G1, S, G2/M phase block. The results of the cell cycle for the cells incubated with Fe_3_O_4_@mSiO_2_-DNM-ZnO NPs or free DNM were given in Figure 6(D). As shown in Figure 6(D), compared with the control, there were significant increased G2/M phases’ distributions in the cell cycles for the cells treated with both free DNM and Fe_3_O_4_@mSiO_2_-DNM-ZnO NPs. Since the G2/M phases represent the DNA synthetic period of the cancer cells, the enhanced distribution in these phases indicated that the antiproliferative mechanism induced by the inhibition of DNA duplication. This result was in high consistent with the well-known conclusion that DNM induced the apoptotic of cancer cells by inserting DNA duplex structures with its plat anthraquinone rings (Lukyanova et al., 2009; Kafri et al., 2013; Nitiss & Nitiss, 2014). For the Fe_3_O_4_@mSiO_2_-DNM-ZnO group, the increased G2/M phase distribution also indicated that the drug loading process did not influence the anti-cancer activity of DNM. The mechanism of cell apoptosis was investigated by the following flow cytometry methods.

## Conclusion

Herein, ZnO QD has been successfully used as an acid-sensitive gatekeeper to block the mesopores of Fe_3_O_4_@mSiO_2_ NPs by physical adsorption. As confirmed by *in vitro* drug release assays, the controlled release of the DNM drug could be achieved in an acidic pH (acidic microenvironment of tumor cells) to dissolve the ZnO QD gatekeepers. Intracellular experimental results proved that DNM-loaded functionalized MMSN not only had enhanced tumor cells inhibitions but could also trace the accumulation of Fe_3_O_4_@mSiO_2_ NPs to monitor the therapy process by the red fluorescence. These results demonstrate that ZnO gatekeepers not only showed well response by the ‘on-off’ switch of the pores but also enhance the anti-cancer effect of Zn^2+^, while ZnO QDs rapidly dissolve in an acidic condition of cancer cells, thus increasing the cancer cell killing efficiency. The ZnO-gated Fe_3_O_4_@mSiO_2_-DNM NPs drug delivery system exhibits prospective applications for cancer theranostics.
